# Viewing patterns regarding panoramic radiographs with different pathological lesions: an eye-tracking study

**DOI:** 10.1259/dmfr.20210019

**Published:** 2021-06-08

**Authors:** Dorothea Vogel, Ralf Schulze

**Affiliations:** 1 Department of Prosthodontics and Materials Science, University Medical Center of the Johannes Gutenberg-University Mainz, Mainz, Germany; 2 Department of Oral and Maxillofacial and Plastic Surgery, University Medical Center of the Johannes Gutenberg-University, Mainz, Germany; 3 Department of Oral Surgery and Stomatology, Division of Oral Diagnostic Science, University of Bern, Bern, Switzerland

**Keywords:** Dental education, Eye-tracking technology, Eye movements, Panoramic radiography, Diagnostic techniques and procedures

## Abstract

**Objective:**

The aim of this study was to examine how dental students vary their viewing patterns of panoramic radiographs during different levels of dental education.

**Methods:**

Two groups of students (total number = 48, n = 24) in different grades (second and fifth clinical semester) were compared. The second clinical semester participated twice, as during the second clinical semester a specific lecture on dental radiology and diagnosis is held. The first viewing took place at the beginning of the semester (2a), the second at the end of it (2e). The fifth semester (5e) represents students shortly before graduation. While viewing 20 panoramic radiographs showing specific pathologies, the eye movement was captured by an eye-tracker. After a maximum of 60 s per image, the students had to report a suspected diagnosis. Every panoramic radiograph included a pathological lesion which was diagnosed by an expert observer who also defined the areas of interest (AOI). The images were presented in the same order to each participant. The metric data recorded by the tracking-system included total time to first fixation, total fixation count, total gaze duration and coordinates of the fixation in and outside an area of interest. In addition, parameters like the completeness of scanning and the suspected diagnosis were analysed. Differences between the groups were assessed for statistical significance and associations between level of different grades, viewing time, completeness of scanning and correctness of diagnosis were computed.

**Results:**

2e was significantly faster (*p* < 0,001), whereas 5e was significantly (*p* < 0.001) more likely to diagnose correctly and also to scan more completely. Scanning duration did not significantly influence the correctness of diagnosis. The lower edges of the panoramic radiographs were not scanned as often as the centre of the image. Bony lesions were generally found to be difficult to interpret and significant findings located in the sinus were overlooked the most.

**Conclusion:**

The higher semester had a more complete viewing pattern and diagnosed correctly with a higher percentage. After hearing the mentioned lecture, the second semester scanned faster and mentioned the AOI more often but could not make a right diagnosis.

## Introduction

In Germany, 40% of radiologic examinations of all healthcare disciplines between 2007 and 2015 were used for dental reasons.^1^ Radiology is fundamental for dental diagnostics and records, especially panoramic radiographs (PAN) are commonly used as they represent a large overview of the dentition, the temporomandibular joint, the lower jaw and parts of the upper jaw including the maxillary sinus.^2^ The broad anatomical coverage of PANs is useful as initial baseline examination, for searching inflammatory focuses, as check-ups of the wisdom teeth, sinus illnesses, traumata, planning and reporting of prosthetic and surgical procedures as well as teeth development and other pathologies.^2–5^ It is essential that dentists know the anatomy and the variables of the projected structures to understand and interpret such images in total and therefore to decide on the right treatment.^6^ Many German dental textbooks describe what aspects and areas on PANs are to evaluate but do not recommend a standardized order to view the image.^6,7^ On the other hand, there are publications that propose a certain search strategy.^8^ In general medicine for instance scanning patterns of radiologists viewing mammograms and computer tomographic images (CT) have been investigated.^9,10^ In dentistry, only a few studies of radiographic images such as PANs or CTs in combination with eye-tracking have been published.^11–17^ In general, it appears that experienced clinicians scanned faster and in a more systematic way but incompletely whereas unexperienced clinicians and students did not seem to follow a viewing scheme.^10,12,13,18^ *Bahazig et al*^14^ recently published that the expert group needed longer examination times on PANs than the group of novices.

Viewing mammograms or CTs differ significantly from viewing PANs. The results of eye-tracking studies in general medicine cannot be compared directly to the ones in dentistry because of their different complexity and diagnostic requirements. On the other hand, the level of experience and efficiency of viewing radiographs seem to be positively correlated.^13^

As localization and correct identification are two quite different matters, eye-tracking in combination with diagnosing could be useful to objectify the study. An image projected on the fovea does not necessarily mean that the observer recognizes and processes this image.^19^ Eye-tracking can be a convenient devise to check if the scanned areas are not only looked at but also if the seen structures are perceived and diagnosed.

Since viewing patterns and strategies in terms of scanning time, completeness and correctness of diagnosis are largely unknown for panoramic radiographs, our study aimed to compare those patterns for two cohorts of student groups at different educational stages. A secondary aim of the study was to possibly conclude on options to improve teaching of a viewing strategy for these particular dental radiographs.

## Methods and materials

### Observers

As participants, two cohorts of each 24 students (female/male ratio: 2:1) of two different clinical semesters were recruited from the Faculty of Dentistry of the Johannes-Gutenberg University of Mainz, Germany. These two semesters were selected to represent different levels of experience. The second semester (fourth year students) had to participate twice to investigate the influence of a basic course (“Specific Dental Radiology and Diagnosis”) in radiographic image viewing and interpretation held during that semester. No specific search strategy or compartmentation of PANs into octants was discussed. The first observation (2a) started at the beginning of that semester. The second observation (2e) was conducted at the end of the second semester. 2a can be regarded as the group of beginners, while 2e represents a basic level of experience after hearing the lecture. The fifth clinical semester (5e, fifth year students) represents the level of knowledge shortly before graduation and, therefore, by the end of the undergraduate educational career.

### Content of the undergraduate student education how to read/interpret radiographs

The German undergraduate curriculum in dental radiography is constructed in a way, that the external radiation protection regulations to achieve a “Fachkunde” (license to justify/acquire and interpret radiographs) are met by the end of the undergraduate study period. This requires at least the documented report of 100 dental radiographs (intraoral, panoramic and cephalometric) in combination with general lectures on how to interpret radiographs and on radiation protection. In every clinical semester (from first to fifth), many radiographs are shown and discussed in various lectures. In Mainz, we also have additional interactive lectures (distributed over two semesters) in which students have to report orally on dental radiographs that are displayed on the screen. It is important to emphasize that according to national regulations, the students are educated to read and interpret the entire radiograph from the very beginning.

### Panoramic radiographs

**Figure 1.**
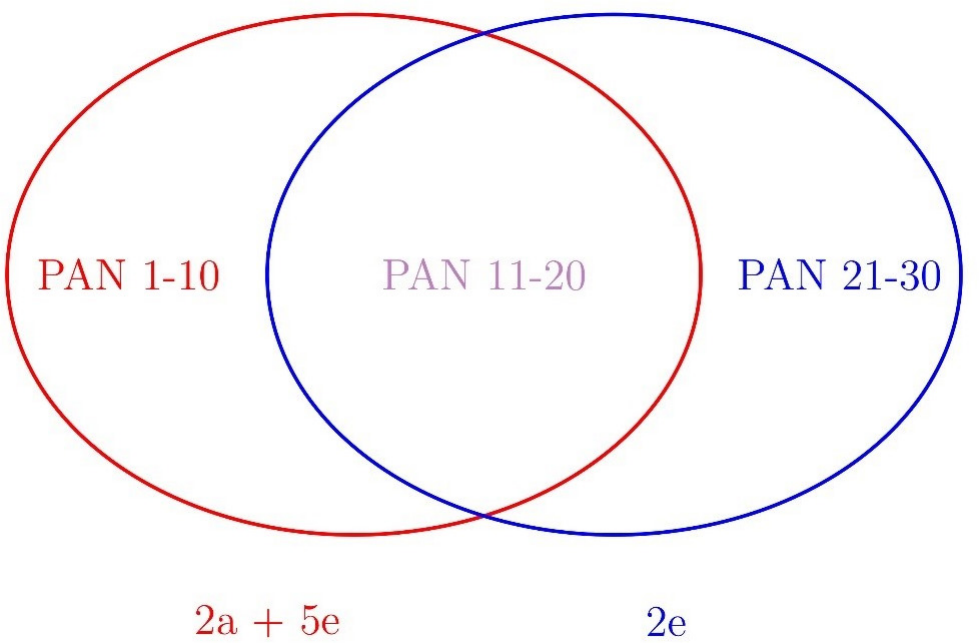
Sketch of the image reading process. Junior students at the beginning of their second clinical semester (2a, fourth year) evaluated PAN 1–20 together with the senior students at the end of their fifth clinical semester (5e, fifth year). Since a subset (1–10) of the PANs 1–20 had been utilized in a radiographic lecture during the second clinical semester, to avoid easy recognition for the 2e-cohort (end of their second clinical semester), these radiographs had been replaced by PANs 21–30, which were solely evaluated by 2e (plus PANs, 11–20), yet did not enter the statistics (explanation see discussion).

In total, 30 radiographs were selected. One tested set consisted of 20 PANs. The second clinical semester at the beginning (2a) and the fifth clinical semester (5e) were shown the same 20 images (Figure 1). As some images were exemplarily used in the lecture mentioned below, these were not shown in the second observation of the second clinical semester (2e) to avoid easy recognition. These radiographs were substituted by an additional set of 10 panoramic radiographs so that 2e reviewed 10 PANs plus viewed ten new ones. The new radiographs were not included into the statistical analysis. Altogether, 1200 evaluations (2a: 24 students *20 PANS +2e: 24 students*10 PANs + 5e: 24 students*20 PANs) were available for statistical analysis.

Initially, each PAN was assessed by an oral radiologist (20 years working experience) who also marked the one or more areas of interest (AOI) on each radiograph (printout version) and defined a suspected diagnosis which served as reference. The AOIs, located in different anatomical structures and divided into categories such as dentition, bone, maxillary sinus and others (soft tissue, tonsils, orbit), were transferred into the eye-tracking software.

**Figure 2.**
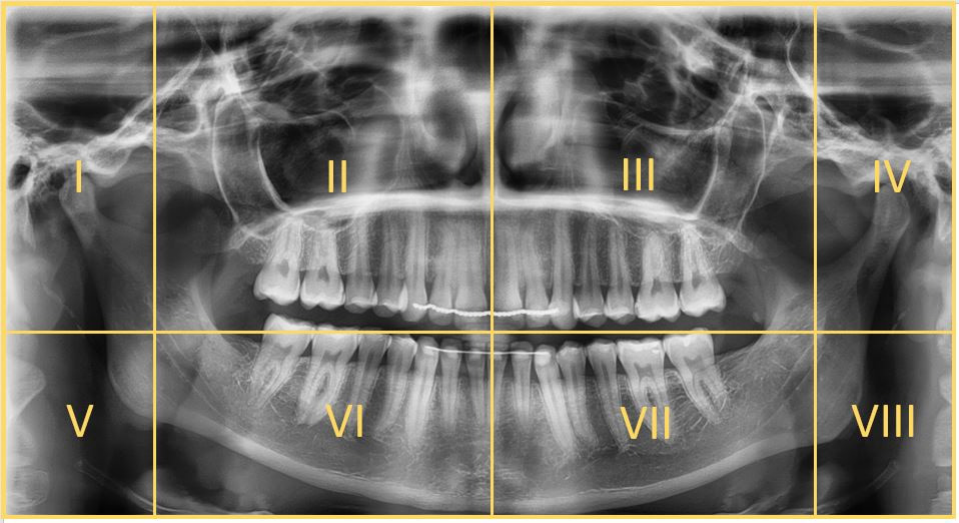
Compartmentation of a panoramic image into octants.

To enable definition of geometric viewing patterns, PANs were divided into octants invisible to the observers (Figure 2). As the locations of the projection of the anatomical structures are different in each image depending on the X-ray machine, the image compartmentation was not of identical size in all PANs. Instead, definition of the octants followed anatomical regions as shown in Figure 2. Consequently, in every image, each octant contained almost the same structures, for example, octant number one always portrayed the right collum mandibulae.

### Viewing of the images

The image evaluation was conducted on a LED monitor (Fujitsu Technology Solutions, Minato, Tokyo, Japan) in a quiet and darkened room with white walls and no distractions in the observer’s field of view. In accordance with the German standard DIN 6868–157,^20^ a daily quality check was performed using the test pattern TG18-QC.^21^ The monitor mounted eye-tracker (EAS Monocular, LC Technologies, Inc., Fairfax, Virginia, US) with the Software Nyan v.2.0 (Interactive Minds GmbH, Dresden, Germany) was used to record each participant’s viewing path. A chin rest was utilized to reduce head movements and secure the distance between participants and the monitor (ca. 60 cm). The observation started by an oral instruction of the participant and a calibration process of the eye-tracker. A calibration consisted of a 15 s lasting nine-point calibration image. The 20 PANs were shown in the same random order to each participant who were also informed that every radiograph contains at least one AOI. The maximum of time per image was 60 s. However, the students could also individually decide to end the assessment earlier by a mouse click. Between the PANs, a black screen was displayed and the participants were asked to describe orally the AOI they had seen and to report a suspected diagnosis. An instructor recorded whether the students named the AOI and which diagnosis they made. No clinical background information on the PANs were given.

Direct parameters were recorded by the eye-tracking software and can be subdivided into those inside AOI and those outside AOI. They included metric data, such as total time to first fixation [s], total fixation count, total gaze duration [s], as well as x/y coordinates of fixation including a time stamp. Total time to first fixation expresses after how many seconds the first fixation in or outside the AOI took place. The total gaze duration quantifies the amount of seconds that a participant spent inside or outside an AOI or in total on the entire slide.

**Figure 3.**
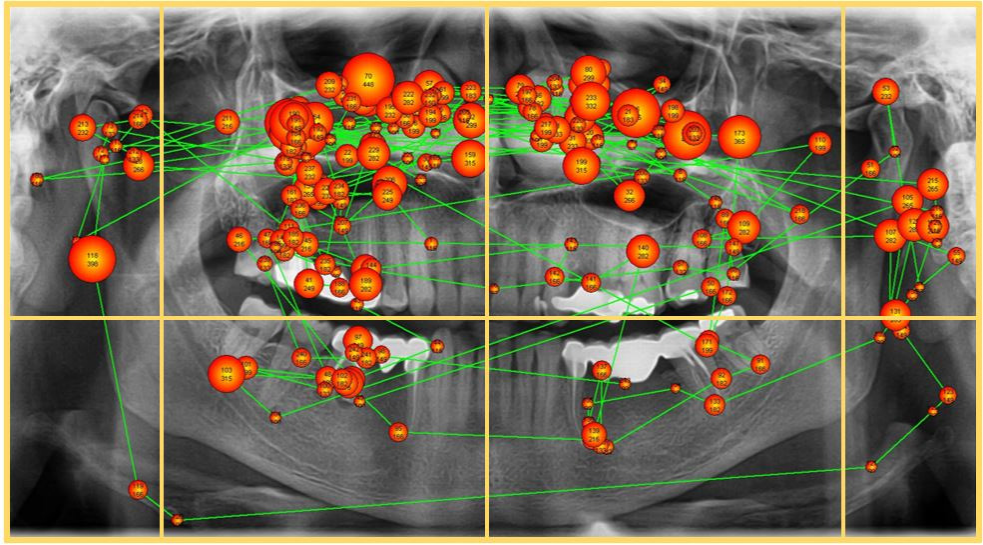
Sample of a complete scan path with compartmentation. Larger points indicate longer viewing times.

Indirect parameters consisted of the completeness of scanning and the correctness of diagnosing. Each participant’s scan path was projected onto the PAN to evaluate which octants the scan path entered. If the scanning entered eight octants, the viewing was considered as complete (Figure 3).

The student’s verbal report for each PAN was analysed and categorized into three diagnosis categories:

- the student’s diagnosis matches the expert’s diagnosis
- the student mentions and describes the AOI with an incorrect/no suspected diagnosis
- the student does not mention the AOI

The tracker-software records heatmaps, defined as data visualization of the focus of visual attention. In order to compare the intensity of viewing between the different groups heatmaps, based on the average fixation duration, were used as color-coded pictures. Two PANs were selected exemplarily to explore the main focus, as they included two different types of pathologies in soft tissues and bone.

### Statistical analysis

Statistical analysis was performed using IBM SPSS (International Business Machines Corporation, Armonk, NY, USA). The data (total time to first fixation, total fixation count, total gaze duration, diagnosing and completeness of scanning) were tested graphically for normal distribution as well as by means of the Kolmogorov–Smirnoff test. Except “total viewing time”, the parameters could be assumed as not normally distributed. Differences between three groups were tested for statistical significance using Kruskal–Wallis test. If the level of significance was α < 0.05, each combination of two groups was analysed using Mann–Whitney U-test. As “total viewing time” was normally distributed, ANOVA and the Bonferroni post-hoc test were applied. A Spearman correlation between completeness of scanning and time was calculated. Charts were prepared.

## Results

The results can be subdivided into temporal viewing behaviour(Table 1) , scanning pattern and diagnosing.

**Table 1.** Results of temporal viewing behaviour

Group	Total time [s]	Total time to first fixation AOI [s]	Total gaze duration AOI [s]	Total gaze duration outside AOI [s]
2a (*n* = 24)	21.04	2.08	2.46	16.25
2e (*n* = 24)	16.2	1.48	2.73	10.97
5e (*n* = 24)	23.62	2.00	2.62	17.26
total (*n* = 72)	21.15	1.93	2.56	15.49

### Temporal viewing behaviour

All PAN assessments were ended before the 60 s time limit. The median value of viewing time was 21.04 s in Group 2a, 16.2 s in Group 2e and 23.62 s in Group 5e. 2e scanned significantly quicker (*p* < 0.001) than 2a and 5e.

As expected, there was a positive correlation (*r* = 0.36) between completeness of scanning and time. The increase was not linear, apparently it did not seem to matter if the students scanned four or five octants.

No significant differences (*p* = 0.381) between viewing time of the images based on correctness of diagnosis were revealed. The participants that diagnosed correctly spent some seconds, but not significantly longer (*p* = 0.381) on the PANs than the ones that did not.

Female observers displayed shorter viewing times than male observers (*p* < 0.001). However whereas the males scanned more completely (*p* = 0.078).

2e spent significantly (*p* < 0.001) less time scanning the part of the PAN outside the AOI (10.97 s) than 5e (17.62 s). 2a spent 16.25 s looking at the PANs outside AOI.

2e (1.48 s) fixated the AOI significantly quicker than 2a (2.08 s; *p* = 0.008) and 5e (2.00 s; *p* = 0.002).

### Scanning patterns

**Figure 4.**
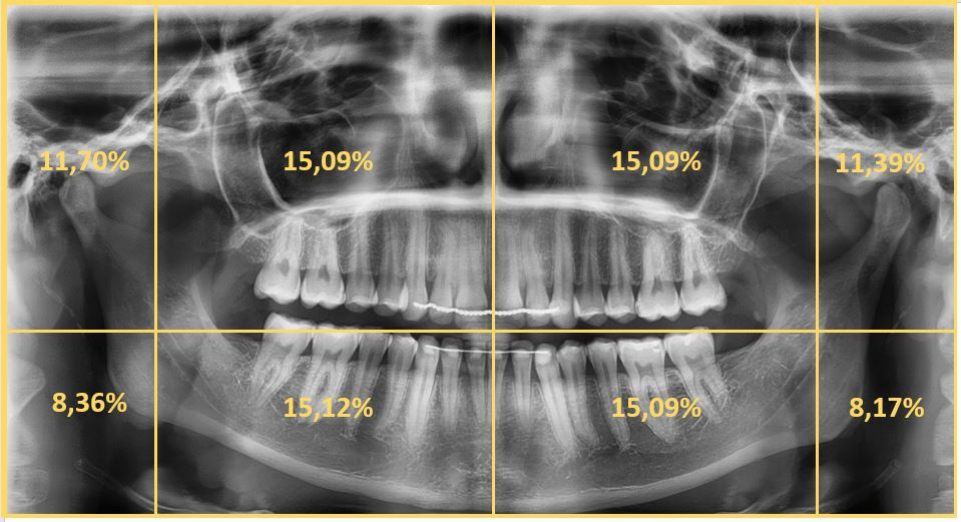
Percentage of viewing of the respective octants pooled over all observers and observations.

The four octants in the centre of the PANs attracted the most attention and were scanned almost equally (Figure 4).

5e on average scanned 46% of the PANs completely, whereas 2a on average scanned 20.8% and 2e 30.8% of the images completely. Since the second semester reduced scanning four or five octants in total by 43%, the scanning of six and eight octants increased.

**Figure 5.**
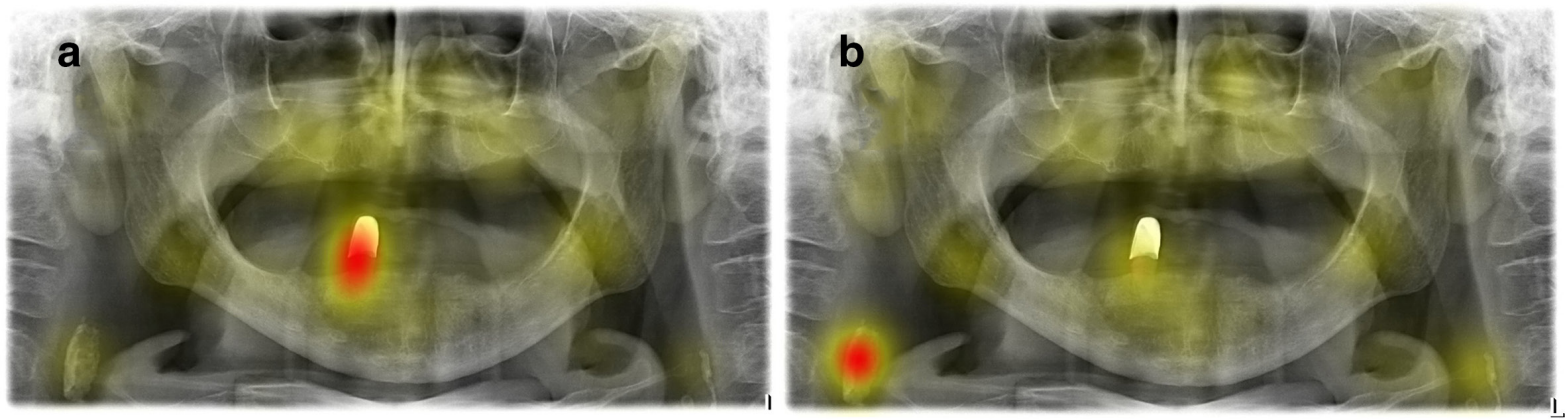
Example of two completely different heatmaps for the same PAN for second (2a, left image a) and fifth semesters (5e, right image b).

Evaluation of the exemplary image revealed that the heatmaps differed largely between the second semester cohort (2a, picture A) and the fifth semester cohorts (Figure 5).

### Diagnostics

In the group of the fifth semester, 35.2% diagnosed the PAN correctly (category I), which differs significantly (*p* < 0.001) from 2a (20.4 %) and 2e (16.7 %). As seen in Table 2, 2e reduced making the correct diagnosis by 18.1%, whereas mentioning the AOI increased by 15.76% and not mentioning the AOI was reduced by 27.18%.

**Table 2.** Diagnosis of the cohorts in percentage

Group	Diagnosis category I	Diagnosis category II	Diagnosis category III
*2a* (*n = 24*)	20.4%	59.0%	20.6%
*2e* (*n = 24*)	16.7%	68.3%	15.0%
*5e* (*n = 24*)	35.2%	57.9%	6.9%
*total* (*n = 72*)	25.6%	60.4%	14.0%

Category I: student’s diagnosis matches expert’s diagnosis, category II: student mentions AOI with incorrect/no suspected diagnosis, category III: student does not mention AOI.

**Figure 6.**
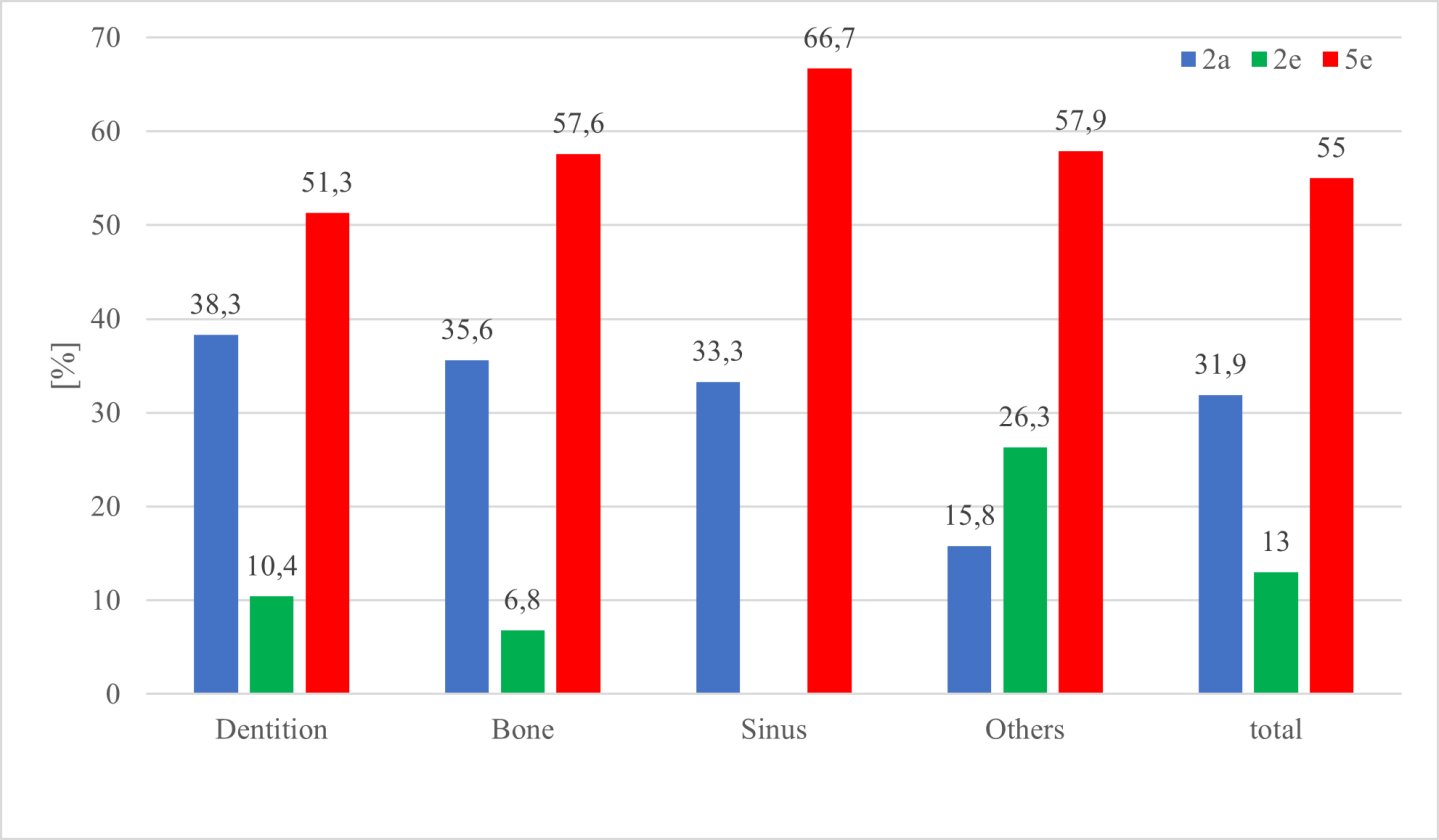
Correct diagnosis (diagnosis category I) of the cohorts in percentage within the localization of the AOI.

**Figure 7.**
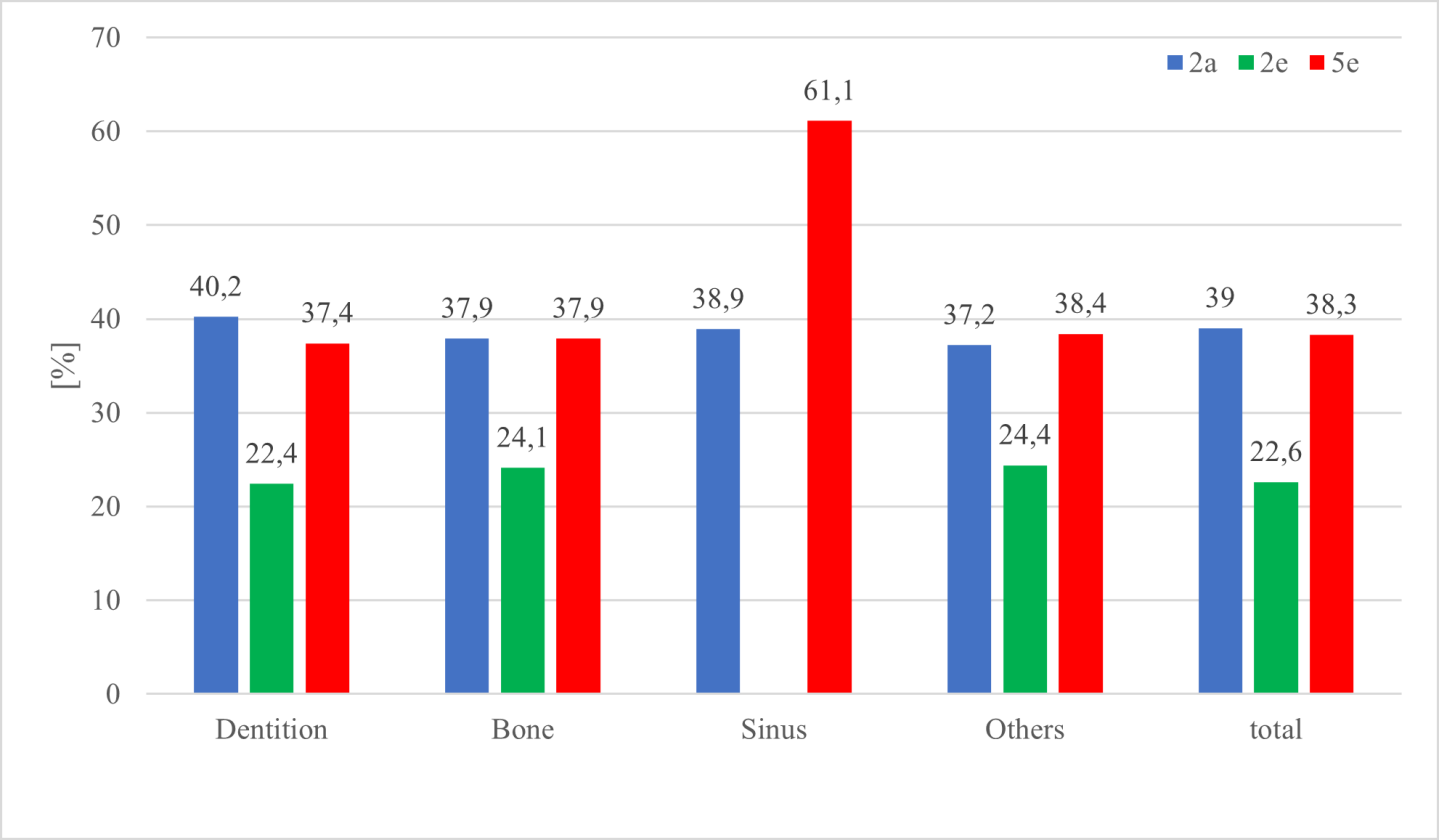
Diagnosis category II (=student mentions AOI with incorrect/no suspected diagnosis) of the cohorts in percentage within the localization of the AOI.

**Figure 8.**
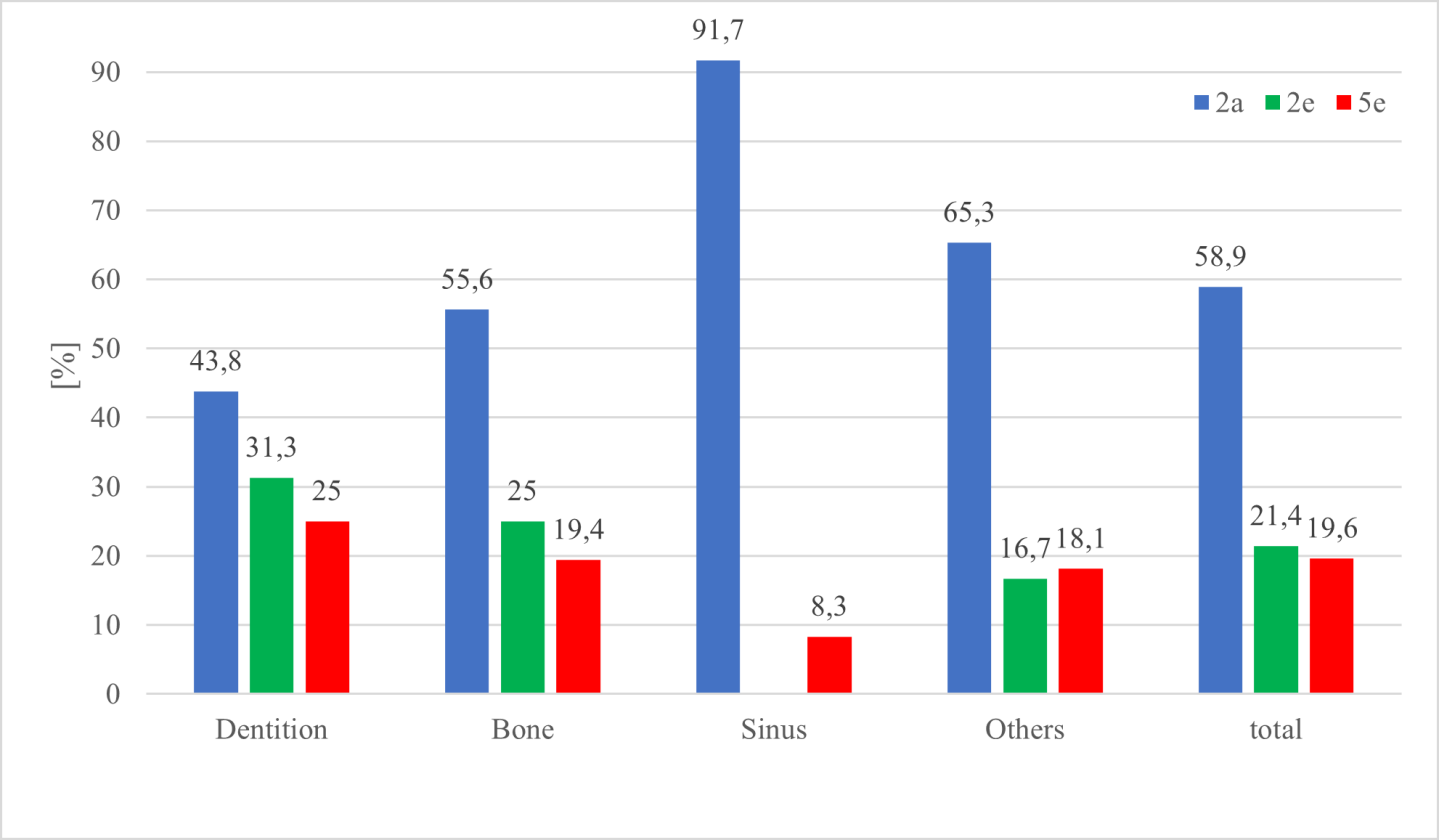
Diagnosis category III (=student does not mention AOI) of the cohorts in percentage within the localization of the AOI.

The distribution between diagnosis, cohorts and localization of AOI is visualised in Table 3 and Figures 6–8. The correctness of diagnosis related to the location of AOI shows that 63% of bony lesions and 62.5% of lesions located in the category "others" were mentioned but not answered (correctly). 307 of the 1200 (25.6%) viewed PANs were diagnosed correctly. 50.2% of the correctly diagnosed pictures contained AOIs in the tooth-bearing area. 58.8% (90 of 153) out of all correctly diagnosed PANs outside the tooth-bearing areas (including bone, maxillary sinus and others) were given by 5e, 25.5% (39 of 153) by 2a and 15.7% (24 of 153) by 2e. 5e had a significantly higher percentage (*p* < 0.001) for diagnosing correctly AOIs in non-tooth-bearing tissue. 2e did not view the PAN with the foreign body in the sinus because it was part of the lecture they received during the monitored semester.

**Table 3.** Diagnosis of the cohorts in percentage within the semester

		Dentition	Bone	Sinus	Others
*Diagnosis category I* *n = 307*	2a	60.2%	21.4%	6,1%	12,20%
	2e	40.0%	10.0%		50.0%
	5e	46.7%	20.1%	7.1%	26.0%
	total	50.2%	19.2%	5.9%	24.8%
*Diagnosis category II* *n = 725*	2a	56.5%	19.4%	2.5%	21.6%
	2e	54.3%	21.3%		24.4%
	5e	53.6%	19.8%	4.0%	22.7%
	total	54.9%	20.0%	2.5%	22.6%
*Diagnosis category III* *n = 168*	2a	21.2%	20.2%	11.1%	47.5%
	2e	41.7%	25.0%		33.3%
	5e	36.4%	21.2%	3.0%	39.4%
	total	28.6%	21.4%	7.1%	42.9%

Category I: student’s diagnosis matches expert’s diagnosis, category II: student mentions AOI with incorrect/no suspected diagnosis, category III: student does not mention AOI. 2e PANs did not contain the radiograph with AOIs in the sinus, therefore the box in the table is empty.

## Discussion

The intention of this study was to compare viewing patterns on panoramic radiographs of rather inexperienced versus slightly more experienced observers. From these differences, we hoped to draw conclusions on learning effects. We used eye-tracking to generate quantitative and qualitative results of how students evaluate panoramic radiographs. This is the main advantage of eye-tracking methods, as otherwise such information is difficult to obtain.

Two different cohorts of students in their clinical semesters were compared for this purpose – one in their second clinical semester (fourth year) and one in their last, *that is*, shortly before graduation (fifth year). We selected panoramic radiographs with specific, relatively typical pathologies as identified and described by an expert observer. Many studies^10,12–16,18,22,23^ have compared experienced versus unexperienced observers in viewing different types of radiographs.

To the best of our knowledge, the present study is one of the first, beside *Bahaziq*,^14^ that analyses a suspected diagnosis in combination with eye-tracking on panoramic radiographs. It also seems to be the first one that compares two different levels during university career.

All in all, we observed an improvement of diagnosing over this undergraduate learning period. Plus, experienced students seem to evaluate the radiographs more completely compared to the less experienced. Authors in^10,12,13^ observed that experienced participants scanned significantly faster. Whereas their viewing process was less complete compared to unexperienced observers. As our students in the unexperienced cohort were just starting to learn about radiographic diagnosis, it may be speculated that they were simply concentrating on the teeth images since this already took all their attention. Neighbouring structures displayed on the radiographs may have simply overburden their limited diagnostic capacity in that early stage of their education.

Khalifa et al^23^ observed that in the group of students, who took part in lessons on how to read a PAN, the viewing time increased in comparison with an untrained group. The authors^23^ concluded that the trained group might have evaluated the radiographs overcautiously. Our results are partly pointing into the opposite direction. The shorter viewing time of the cohort at the end of the second clinical semester (2e) could be explained by the lack of experience and concentration on well-known structures (*e.g.* teeth). In concordance with the findings of *Khalifa,*^23^ the best-trained group (5e) spent more time on scanning the images.

The positive correlation between viewing time and completeness indicates that with increasing time the participants may ensure not to miss a significant finding. The more complete scan of this cohort is also probably a result of education level. During their clinical education, dental students in our school learn to search for pathologies outside the dental tissue. This can be shown exemplarily comparing the heatmaps of cohort 2a and 5e (Figure 5). These indicate that the main attention changed from the single tooth in the fourth quadrant to the atherosclerotic plaque in the right carotid artery. Similarily to the finding in the study of *Hollevoet et al*^13^, the lateral part of the PAN and, therefore, the soft tissues and part of the spine attracted less attention compared to the teeth and the jaws. Nevertheless, an improvement regarding the completeness of reading over educational time was observed in the present study.

Interestingly, a study by Grünheid et al^12,13^ revealed that new clinicians scanned significantly more completely than a group of experienced orthodontists who missed 75% of significant findings. The explanation of the authors was that the specialists only concentrate on the area of their expertise. It was suspected that radiologists in general medicine may analyse radiographs more neutrally as they do not focus on a specific region. Obviously, it is essential to view and diagnose the entire image. Clinicians that used a “dental only” search scheme did not fixate sufficient areas of interest and, therefore, tended to miss significant findings.^12,13^

It may be important to teach students a standardized scan scheme of panoramic radiographs to ensure that at least the entire radiograph is being read. One simple suggestion would be to view all eight “octants” of the PANs in a subsequent order. A possible way could be scanning octant one to eight and interpreting the image afterwards. We do not have evidence for this strategy yet. Hopefully, this could reduce the number of missed significant findings in the images and counteract a satisfaction of search, which means that the observer stops analyzing the image as soon as an area of interest is found.^15,24^ *Perschbacher*^8^ recommended starting with osseous formations and surrounding non-tooth-bearing areas, continuing with the alveolar processes and finally the teeth. Of course, both strategies require teaching of the relevant structures that are commonly displayed in certain areas. Viewing is one requirement, interpreting and diagnosing another.

Previous studies^25,26^ mention an error range from 2 to 27% in making the right diagnosis. *Bahaziq et al*^14^ reported that in the group of novices as well as in the group of experts (orthodontists), the detection skills were better than the interpretation skills. At the same time, it is expected that with increasing experience and practice the diagnosing capabilities will enhance.^6^ Interestingly, we observed that bony defects were frequently mentioned by the students but could not be diagnosed correctly or even no differential diagnosis was provided. We conclude that such lesions in the bone seem to pose special challenges on interpretation.

There were obviously some limiting factors in this study. For instance, we only received a verbally expressed diagnosis owning the fact that the observer was fixed on the chin rest to remain within the calibrated area of the device. Nevertheless, we observed some minor motion of the observers during the evaluations. Although this cannot be easily avoided, it may also have influenced the accuracy of the eye-tracking data. Another limitation was the knowledge of the participants that every image contained at least one significant finding or pathology. Hence, the students were expecting to find and thus presumably searching for the AOIs. This probably influenced their search pattern. The participants were given a time limit of 60 s for every PAN to limit the overall time for the study per observer. From retrospect, we assume that for unexperienced observers one minute for analysing a PAN is possibly too short. On the other hand, we initially intended to avoid lengthy observation periods for the entire set of images since we expected considerable loss of attention. Windowing or levelling of the images was not allowed, due to the possible uniform comparison of the eye-tracking data. This does not really reflect the ideal viewing scenario; however, it may reflect typical viewing situations in clinical applications where certainly windowing and levelling is also not commonly applied. Another limitation was the assumption of a complete scan path when the path entered all eight octants even if there were only some fixation points per octant as seen in Figure 3.

Also, from our study, we cannot directly conclude on skills development of individual students as the individuals within the cohorts were different. If assuming some representativity of the 24 student cohorts, however, the general conclusions of a more complete evaluation of radiographs plus a slightly more “accurate” diagnosis may still be drawn.

Another drawback was the statistical evaluation of only 10 out of the 20 PANs evaluated by cohort 2e, which certainly introduces some statistical bias due to the smaller sample size. As to the same amount of PANs and, therefore, same attention span of 10 new PANs were substituted for the second viewing of the second semester (2e).

Considering our outcome and the shortcomings involved with the study, future studies are encouraged using the eye-tracking technique for different types of dental radiographs. A good idea would be to monitor one cohort over time, that is, from the second to the fifth clinical semester. It should be noted here that eye-tracking is hardly practical for dynamically evaluated images such as CBCT. To evaluate such dynamic viewing, a quicker eye-tracking system capable to track the fixation points for each slice of the 3d-dataset would be required.

## Conclusion

Within the limitations of this study, we observed a more complete diagnosing of panoramic radiographs towards the end of the undergraduate study period. Evaluations of late semesters were much more complete than those of the earlier cohort even if the main focus still remained in the centre (*i.e.* the tooth plus the teeth-bearing tissue) of the PAN. It may be speculated if a standardized method for analysing PANs, for example, scanning all octants in a certain order and afterwards interpreting noticeable structures, could result in a more complete evaluation of those dental radiographs.
